# Effects of the Use of Assisted Reproductive Technologies and an Obesogenic Environment on Resistance Artery Function and Diabetes Biomarkers in Mice Offspring

**DOI:** 10.1371/journal.pone.0112651

**Published:** 2014-11-11

**Authors:** Francisco I. Ramirez-Perez, Angela L. Schenewerk, Katy L. Coffman, Christopher Foote, Tieming Ji, Rocio M. Rivera, Luis A. Martinez-Lemus

**Affiliations:** 1 Dalton Cardiovascular Research Center, University of Missouri, Columbia, Missouri, 65211, United States of America; 2 Department of Biological Engineering, University of Missouri, Columbia, Missouri, 65211, United States of America; 3 Division of Animal Sciences, University of Missouri, Columbia, Missouri, 65211, United States of America; 4 Department of Statistics, University of Missouri, Columbia, Missouri, 65211, United States of America; 5 Department of Medical Pharmacology and Physiology, University of Missouri, Columbia, Missouri, 65211, United States of America; Max-Delbrück Center for Molecular Medicine (MDC), Germany

## Abstract

Maternal obesity affects the incidence of cardiovascular disease and diabetes in offspring. Also the use of assisted reproductive technologies (ART) has been associated with cardiovascular deficiencies in offspring. Obese women often suffer from infertility and use ART to achieve a pregnancy, but the combined effects of maternal obesity and ART on cardiovascular health and incidence of diabetes in the offspring is not known. Here, we report the effects of the use of ART within an obesogenic environment, consisting of feeding a western diet (WD) to dams and offspring, on resistance artery function and presence of diabetes biomarkers in juvenile mice offspring. Our results indicate that WD and ART interacted to induce endothelial dysfunction in mesenteric resistance arteries isolated from 7-week-old mice offspring. This was determined by presence of a reduced acetylcholine-induced dilation compared to controls. The arteries from these WD-ART mice also had greater wall cross-sectional areas and wall to lumen ratios indicative of vascular hypertrophic remodeling. Of the diabetes biomarkers measured, only resistin was affected by a WD×ART interaction. Serum resistin was significantly greater in WD-ART offspring compared to controls. Diet and sex effects were observed in other diabetes biomarkers. Our conclusion is that in mice the use of ART within an obesogenic environment interacts to favor the development of endothelial dysfunction in the resistance arteries of juvenile offspring, while having marginal effects on diabetes biomarkers.

## Introduction

Obesity has become a major health problem worldwide [1], [2]. Excessive body weight in the form of accumulated adipose tissue is accompanied by adverse pathophysiological conditions. Of these conditions, hypertension and type 2 diabetes mellitus (T2DM) are particularly important for their association with cardiovascular disease and the incidence of life threatening cardiovascular events such as myocardial infarction and stroke [1]. The epidemic proportions at which obesity is increasing are associated with an increased consumption of hypercaloric diets rich in fats and carbohydrates, as well as with more sedentary lifestyles [3], [4], [5]. In addition to these factors, evidence is increasing that indicates exposure to an obesogenic environment during embryonic and fetal development programs individuals for obesity, hypertension and T2DM later in life [6], [7], [8], [9], [10], [11], [12], [13]. This developmental programming of adult diseases is hypothesized to participate in perpetuating the incidence of obesity and cardiovascular disease across generations [5].

An additional consequence of the obesity epidemic is that many women of reproductive age are overweight and have difficulties getting pregnant [14]. Many of these women pursue the use of assisted reproductive technologies (ART) to address their infertility. The use of ART has been associated with an increased incidence of developmental abnormalities including cardiovascular malformations that is further increased when mothers are obese [15], [16], [17]. This suggests that ART has the potential of influencing the developmental programming of adult diseases. However, little is known about the interactions that ART and maternal obesity may have on the development of adult diseases in offspring.

In order to emulate the increasing association between the use of ART and obesity in women with the incidence of obesity, hypertension and T2DM in the offspring, we recently developed a mouse model of this condition [18]. We have already reported that, in this model, consumption of a western diet (WD) high in fat and carbohydrates by both mother and offspring resulted in augmented mean arterial pressure at seven weeks of age. We also reported that the use of ART resulted in increased body weight at weaning, as well as in the presence of an increased production of reactive oxygen species (ROS) by the resistance arteries of the juvenile offspring [18].

Presence of an increased production of ROS or an imbalance in the redox status of tissues can cause oxidative stress, which in turn has been associated with vascular dysfunction and the development of T2DM [19], [20]. In particular, ROS can cause vascular endothelial dysfunction, which is associated with decreased vasodilation efficiency, a hallmark of both hypertension and diabetes. An increased production of ROS, in conjunction with obesity, is indicative of a pro-inflammatory state that has been associated with changes in biomarkers related to orexia, glucose metabolism and the development of T2DM. In the present study we investigated the effects of the use of ART in the context of an obesogenic environment from embryonic development to early life on the vasoconstriction and vasodilation properties of mesenteric resistance arteries, as well as on the presence of T2DM associated biomarkers in juvenile mice. We report that a significant interaction exists between an obesogenic environment and the use of ART that affects the endothelial function and structure of mesenteric resistance arteries, while having only a small effect on T2DM associated biomarkers in juvenile mice offspring.

## Methods

### Ethics Statement

All animal procedures were performed in accordance to the Guide for the Care and Use of Laboratory Animals published by the U.S. National Institutes of Health. The Institutional Animal Care and Use Committee at the University of Missouri approved all animal procedures used in the study. Animal use protocol was 7501.

### Mice and ART

**Figure 1:**
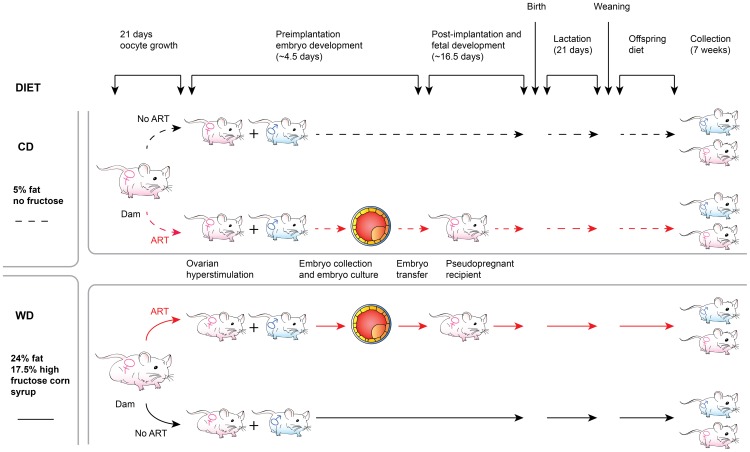
Diagrammatic representation of the experimental design. The design consisted of a factorial arrangement of diet effects (CD = Control Diet; WD = Western Diet), the effects of the use of assisted reproductive technologies (ART; No ART), and sex (males; females) on juvenile mice offspring.

Female mice used for reproductive purposes were of the NSA strain (CF1; Harlan Laboratories, Indianapolis, IN, USA). Males used for reproductive purposes were B6D2F1/J mice (The Jackson Laboratory, Bar Harbor, ME, USA). Dams were split in two groups to receive different diets beginning three weeks before mating with males. The control diet (CD) group of dams was fed chow containing 6.5% fat (LabDiet 5008). The western diet (WD) group received feed containing 24% fat and 17% high fructose corn syrup (TestDiet 58Y1, St. Louis, MO, USA) as previously described [18]. Each group of dams was divided into two subgroups. In the No ART subgroup, dams were mated naturally and allowed to carry their pregnancies from beginning to end. In the ART subgroup, female mice 6–10 weeks old were superovulated with an intraperitoneal injection of equine chorionic gonadotropin (5 IU) followed by human chorionic gonadotropin (5 IU) 45 hours later. Embryo donors were mated with intact males, while embryo recipient dams (surrogates) were mated with vasectomized males. Two-cell embryos were collected 46 hours following superovulation from the embryo donors and cultured in Whitten's medium for three days. Cultured embryos where then transferred to 2.5 days pseudopregnant recipients that received ten embryos per uterine horn. Pseudopregnant surrogate dams were fed the same diet as the embryo donors. We purposely chose to use Whitten's medium for embryo culture because this medium is known to provide suboptimal conditions that affect the molecular structure of the embryo [21], [22], but supports full development to term [18], [23], [24]. In order to equalize litter sizes, pups were culled to constitute litters of four males and four females whenever possible. After weaning, mice were fed the same diet as their mothers and were euthanized at 7 weeks of age. We developed this protocol to emulate, as much as possible, those conditions found in human subjects, in that a woman undergoing ART is usually the recipient of her own embryos and children remain in the same environment after birth [25], [26]. Thus, our experimental design created eight groups of offspring, namely, CD-No ART-males, CD-No ART-females, CD-ART-males, CD-ART-females, WD-No ART-males, WD-No ART-females, WD-ART-males and WD-ART-females (Figure 1). The distribution of offspring per surrogate dam is shown in Table S1. As we are particularly interested in the mechanisms that program offspring exposed to ART and/or an obesogenic environment to hypertension and T2DM early in life, we euthanized mice at an age comparable to human puberty.

### Vessel Isolation

Mesenteric arteries were collected immediately after euthanasia at 7 weeks of age. Two arteries were isolated from each mouse. The arteries were cannulated onto glass micropipettes, pressurized at 70 mmHg without flow, and warmed to 37°C in commercial myograph chambers (Living Systems Instrumentation, Burlington, VT, USA) as previously described [27]. The chambers contained physiological saline solution (PSS) with (in mM) 145.0 NaCl, 4.7 KCL, 2.0 CaCl_2_, 1.2 MgSO_4_, 1.0 NaH_2_PO_4_, 5.0 dextrose, 3.0 3-(N-morpholino) propanesulfonic acid (MOPS), 2.0 pyruvate, and 0.02 EDTA at a pH of 7.4. The micropipettes were filled with PSS that contained 0.15 mM bovine serum albumin. To test for viability, cannulated arteries were allowed to stabilize for 40 min and then exposed to PSS in which NaCl was equimolarly substituted with 80 mM KCl. Only arteries that constricted more than 20% to this 80 mM K^+^ solution were used in the analyses.

After the exposure to high K^+^, the arteries were washed three times with fresh PSS and exposed to increasing concentrations of phenylephrine (10^−8^ to 10^−4^ M) to examine adrenergic-dependent vasoconstriction. Subsequently, arteries were washed and pre-constricted with 10^−6^ M phenylephrine. The arteries were then exposed to increasing concentrations of acetylcholine (10^−9^ to 10^−5^ M). After washing these agents from the arteries, they were again pre-constricted with 10^−6^ M phenylephrine and exposed to increasing concentrations of sodium nitroprusside (SNP, 10^−8^ to 10^−4^ M). All vasoactive agents were added in a cumulative fashion to the bath solution at increments of 0.5 M. Each concentration was maintained in the bath for 2 minutes. At the end of each experiment arteries were exposed to Ca^2+^-free PSS with 2 mM EGTA and 10^−4^ M adenosine to obtain maximal passive diameter. Throughout the experiment, chambers were mounted on inverted microscopes with CCD cameras. Luminal diameter and wall thicknesses were recorded using a video caliper (Living Systems Instrumentation, Burlington, VT, USA) and a Powerlab data acquisition system (ADInstruments Inc, Colorado Springs, CO, USA).

The elastic characteristics of the arteries were determined at the end of each experiment. Vessels were exposed to consecutive changes in intraluminal pressure from 70 mmHg to 5, 10, 20, 30, 40, 60, 80, 100, 120, and back 70 mmHg while under passive conditions (Ca^2+^-free PSS). Each pressure was maintained for 2 minutes while luminal diameter and wall thicknesses were recorded.

### Plasma measurement of diabetes associated biomarkers

At euthanasia, blood from non-fasted mice was collected by cardiac puncture. Blood was allowed to clot and the plasma was collected and centrifuged at 3,000×g for 15 minutes at 4°C to separate any cellular debris from the plasma. Plasma samples were frozen and kept at −70°C for subsequent analyses.

A volume of 50 µl of plasma from each mouse was used to measure the concentration of ghrelin, glucose-dependent insulinotropic polypeptide (GIP), glucagon-like peptide-1 (GLP-1), glucagon, insulin, leptin, plasminogen activator inhibitor-1 (PAI-1), and resistin. The concentration of these biomarkers was measured using the Bio-Plex Pro Mouse Diabetes 8-Plex Assay Kit from Bio-Rad. Plasma samples were analyzed in a BioPlex 200 following instructions from the manufacturer.

### Data Analysis

Luminal diameters of arteries were normalized to maximal passive diameter or the level of initial vasoconstriction (phenylephrine-induced pre-constriction). The normalized data were then regressed to ART, diet, sex, intraluminal pressure, and their interactions. Important fixed factors were selected by controlling the statistical significance at level 0.10. According to experimental design, the offspring effect and mother effect were also included as random effects to incorporate covariances among observations. Correlated repeated measurements on the same experimental unit (each artery) were modeled by several different variance-covariance structures, and the best was chosen based on AIC (Akaike information criterion) and BIC (Bayesian information criterion) as well as biological interpretation. Models were implemented in SAS (version 9.4). Residual plots were carefully examined to check model assumptions. Proper Box-Cox transformations were applied to data to maintain normality and constant variance assumptions. Tukey-Kramer HSD (Honestly Significant Difference) test was applied to adjust multiple tests in pair wise comparisons. Statistical significance was considered at P≤0.05. All data are presented as means ± SEM. An extended version of the statistical analysis is included in Methods S1.

## Results

### Vasoconstriction to phenylephrine was greater in males than in females

**Figure 2:**
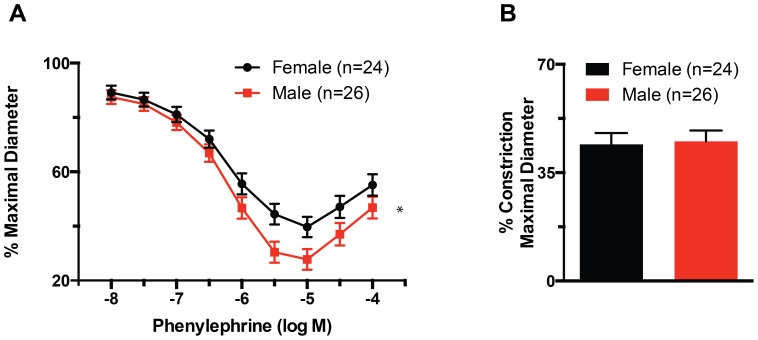
Phenylephrine-induced vasoconstriction is greater in male than in female mice. (A) Percent maximal diameter of mesenteric resistance arteries isolated from male and female mice, and exposed to incremental concentrations of phenylephrine. The maximal constriction to phenylephrine was significantly greater (*P≤0.05) in males than females. (B) Percent constriction after exposure to 80 mM KCl of mesenteric resistance arteries isolated from male and female mice. Data are means ± SEM.

No significant effects were found for diet, ART or their interaction with regard to maximal phenylephrine contraction or the half maximal concentration (EC50) of this vasoconstrictor. The only significant effect found for the vasoconstriction induced by increasing concentrations of phenylephrine was that of sex. Maximal constriction was achieved at a concentration of 10^−5^ M phenylephrine and was significantly (P≤0.05) greater in males than in females (Figure 2A). Exposure of vessels to concentrations of phenylephrine greater than 10^−5^ M was associated with paradoxical reductions in maximal constriction. In comparison, no significant differences were observed between arteries from males or females on the constriction caused by KCl-induced depolarization (Figure 2B).

### Acetylcholine-induced vasodilation was significantly affected by sex and diet x ART interaction

**Figure 3:**
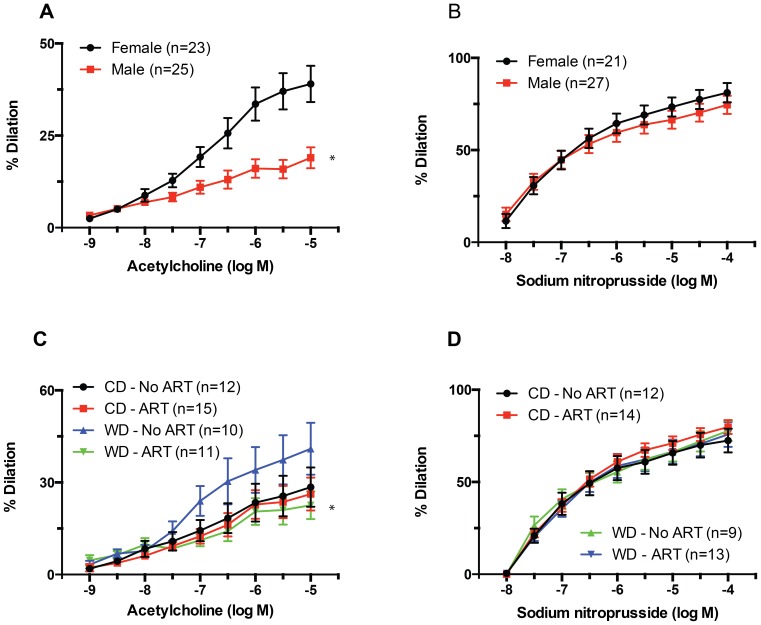
Effects of sex and diet x ART interaction on relaxation responses of mesenteric resistance arteries. (A) Percent dilation of mesenteric resistance arteries isolated from male and female mice, and exposed to incremental concentrations of acetylcholine. Maximal dilations to acetylcholine were significantly greater (*P≤0.05) in females than in males. (B) Percent dilation of mesenteric resistance arteries isolated from male and female mice, and exposed to incremental concentrations of SNP. (C) Percent dilation of mesenteric resistance arteries isolated from mice fed a WD or CD and obtained by natural birth (No ART) or the use of ART, and exposed to incremental concentrations of acetylcholine. Maximal dilations to acetylcholine were significantly smaller (*P≤0.05) in arteries from WD-ART mice than in those from WD-No ART mice. (D) Percent dilation of mesenteric resistance arteries isolated from mice fed a WD or CD and obtained by natural birth (No ART) or the use of ART, and exposed to incremental concentrations of SNP. Data are means ± SEM.

Acetylcholine-induced vasodilation was significantly (P≤0.05) greater in mesenteric arteries isolated from females than in those isolated from males (Figure 3A). This was particularly noticeable at an acetylcholine concentration of 10^−6.5^ M. Acetylcholine-induced dilation was also significantly affected (P≤0.05) by the interaction between diet and ART (Figure 3C). Mesenteric resistance arteries from both males and females in the WD-No ART group had greater maximal vasodilations to acetylcholine than those in the WD-ART group. In comparison, no differences in maximal acetylcholine-induced vasodilation were observed between the CD-ART and CD-No ART groups. Vasodilatory responses to SNP were not significantly different between any of the groups (Figure 3B,D)

### Effects of diet and ART on vascular structure

**Figure 4:**
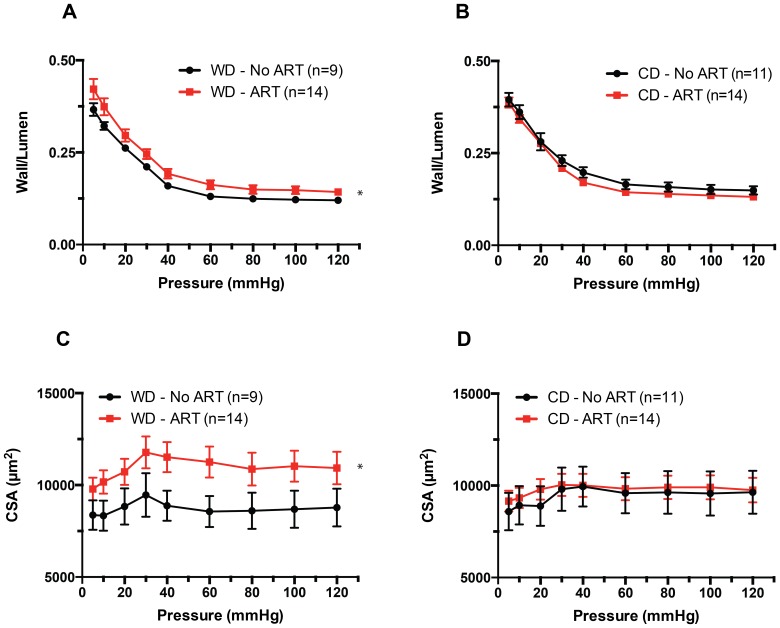
Effects of diet x ART interaction on arterial wall-to-lumen ratios and wall cross-sectional areas. (A) Wall to lumen ratios obtained under passive conditions (Calcium-free) and at different intravascular pressures in mesenteric resistance arteries isolated from mice fed a WD or CD and obtained by natural birth (No ART) or the use of ART. Wall to lumen ratios were significantly greater (*P≤0.05) in ART-WD vs. No ART-WD arteries. (B) Wall cross-sectional areas (CSA) obtained under passive conditions (Calcium-free) and at different intravascular pressures in mesenteric resistance arteries isolated from mice fed a WD or CD and obtained by natural birth (No ART) or the use of ART. CSA were significantly greater (*P≤0.05) in ART-WD vs. No ART-WD arteries. Data are means ± SEM.

A significant effect (P≤0.05) was found for the interaction between diet and ART on the media to lumen ratios (Figure 4A), and vascular wall cross-sectional areas (Figure 4C) of mouse mesenteric resistance arteries maintained under passive conditions. Arteries obtained from ART mice had greater wall to lumen ratios and wall cross-sectional areas than those obtained from No ART mice only when the animals were fed a WD. No differences between ART and No ART were observed in arteries from mice fed CD (Figure 4B,D). Not significant differences were observed either for the internal passive diameter of the arteries or their elastic characteristics, including stress-strain relationships and Young's modulus of elasticity between any of the groups (Figure S1).

### Diet had a significant effect on most of the diabetes related biomarkers measured

**Figure 5:**
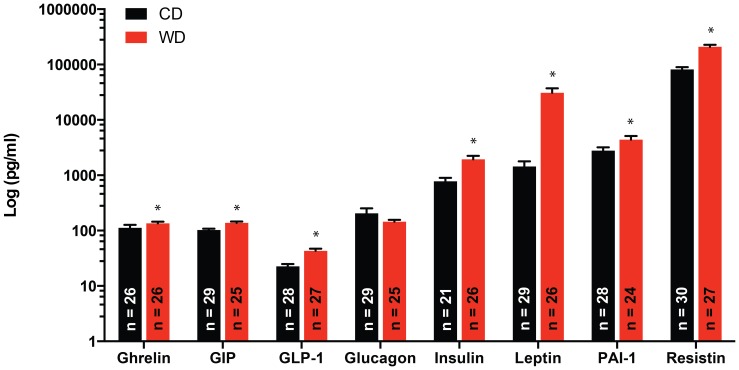
Effect of diet on the serum concentrations of diabetes biomarkers. Serum concentrations of ghrelin, GIP, GLP-1, glucagon, insulin, leptin, PAI-1 and resistin in mice fed a WD or CD. Serum concentrations of ghrelin, GIP, GLP-1, insulin, leptin, PAI-1 and resistin were significantly greater (*P≤0.05) in WD vs. CD fed mice. Data are means ± SEM.

The concentrations of ghrelin, GIP, GLP-1, insulin, leptin, and resistin were all significantly greater (P≤0.05) in serum collected from mice in the WD group vs. the CD group irrespective of sex or ART status (Figure 5). Glucagon was the only biomarker measured that was not significantly affected by diet.

### Effects of sex on the diabetes related biomarkers measured

**Figure 6:**
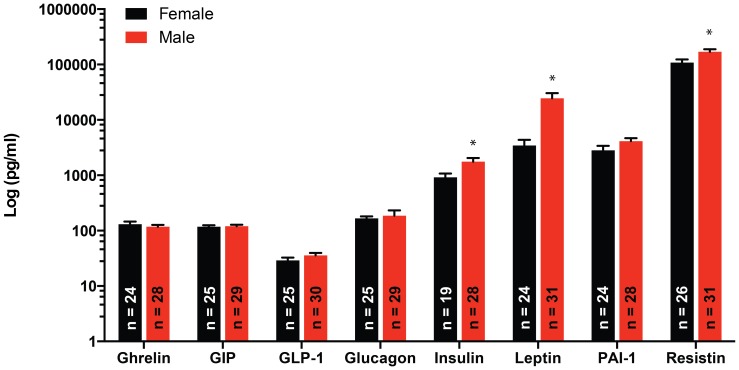
Effects of sex on the serum concentrations of diabetes biomarkers. Serum concentrations of ghrelin, GIP, GLP-1, glucagon, insulin, leptin, PAI-1 and resistin in male and female mice. Serum concentrations of insulin, leptin, and resistin were significantly greater (*P≤0.05) in male vs. female mice. Data are means ± SEM.

Sex had a significant effect (P≤0.05) on the concentrations of insulin, leptin, and resistin, with males having consistently greater levels of these biomarkers in serum (Figure 6).

### Leptin and PAI-1 were significantly affected by a diet x sex interaction

**Figure 7:**
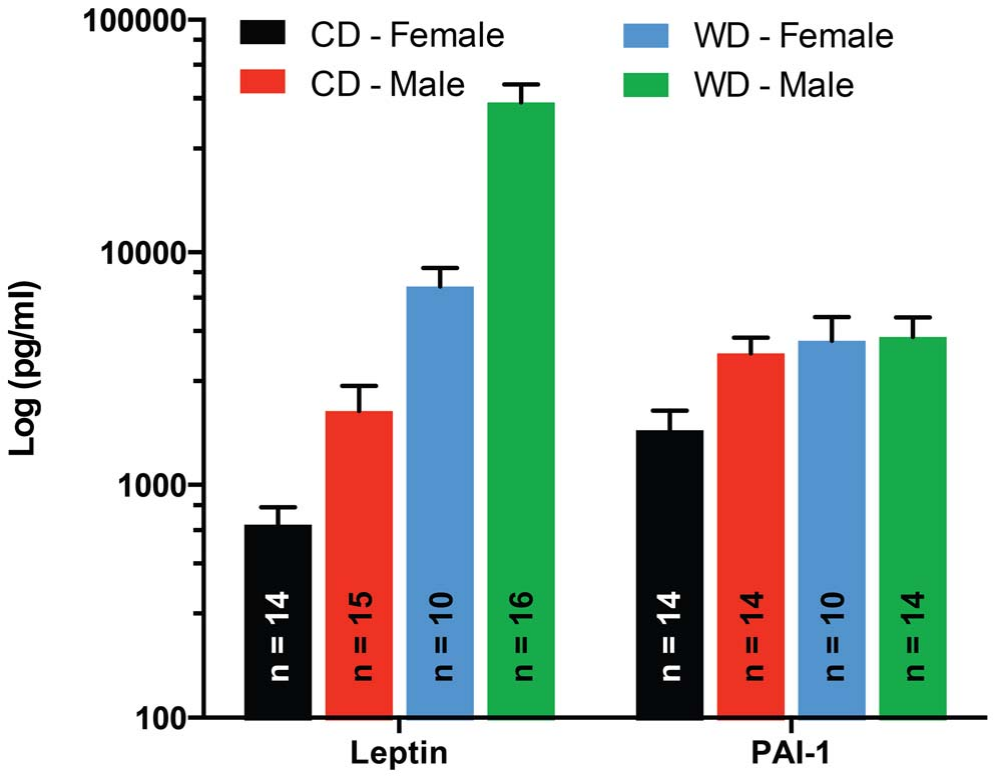
Effects of diet x sex interaction on the serum concentrations of diabetes biomarkers. Serum concentrations of leptin and PAI-1 in male or female mice fed a WD or CD. The increase in serum concentrations of leptin associated with consumption of WD was greater (P≤0.05) in male than female mice, while WD increased (P≤0.05) PAI-1 concentrations in females, but not in male mice. Data are means ± SEM.

The interaction of diet x sex on leptin serum concentrations was manifested as a significantly greater difference between males and females when mice were fed the WD (Figure 7). In comparison, PAI-1 levels in serum were significantly lower in females than in males only in mice fed CD (Figure 7). No significant differences in PAI-1 serum levels were observed between sexes of mice fed WD. Consequently, WD increased serum levels of PAI-1 only in female mice.

### Resistin was significantly affected by a diet x ART interaction

**Figure 8:**
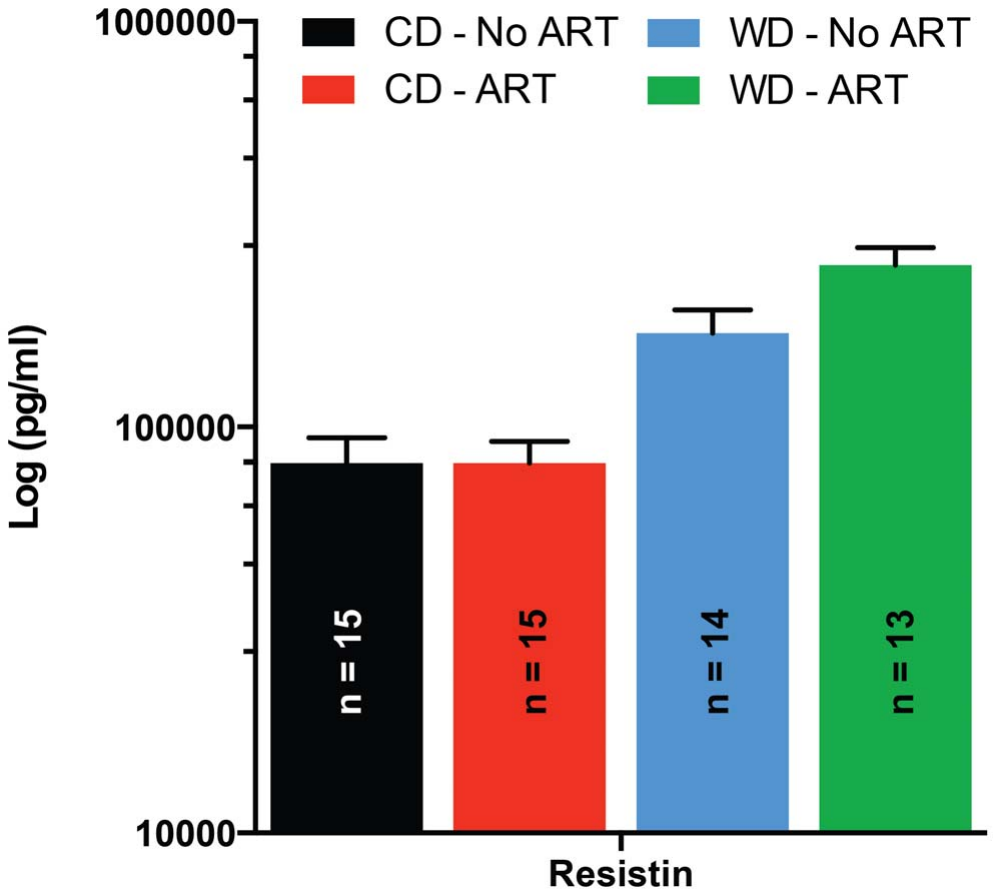
Effects of the diet x ART interaction on the serum concentrations of diabetes biomarkers. Serum concentrations of resistin in mice fed a WD or CD and obtained by natural birth (No ART) or the use of ART. The increase in serum concentrations of resistin associated with consumption of WD was greater (P≤0.05) in ART than in No ART mice. Data are means ± SEM.

As mentioned above, WD increased overall resistin levels, but the increase was significantly greater in ART mice than in No ART mice, resulting in a significant (P≤0.05) diet x ART interaction (Figure 8). A trend interaction (P = 0.06) in PAI-1 serum levels was also observed between diet and ART. PAI-1 tended to be increased in ART mice only when fed a WD. No effect of diet on PAI-1 was observed in mice of the No ART group (Figure S2).

## Discussion

The primary finding of the present study is that the use of ART in an obesogenic environment is associated with a reduced acetylcholine-induce dilation of mesenteric resistance arteries in juvenile mice offspring. We previously reported that the mesenteric resistance arteries of these same mice had an increased level of reactive oxygen species (ROS) in the vascular wall, independent of diet or sex effects [18]. Our present results suggest that exposure to an obesogenic environment tends to increase the ability of mesenteric resistance arteries to dilate more in response to endothelium-dependent acetylcholine stimulation, and that the use of ART under these obesogenic conditions causes endothelial dysfunction. Because ROS reacts with nitric oxide (NO) and reduces its bioavailability, it is likely that the endothelial dysfunction we observed is associated with the well-known scavenging of NO by ROS [12], [28]. We observed a numerical reduction in the transcription level of superoxide dismutase 1 (*Sod1*) in the blood vessels of ART mice (Figure S3). Therefore, it is possible that in the resistance arteries of No ART mice there are sufficient antioxidant mediators to prevent the manifestation of endothelial dysfunction, while in the ART mice even a marginal reduction in oxidant defenses allow ROS to reduce the bioavailability of NO, but this remains to be experimentally corroborated.

Our current results also indicate that the interaction of ART with an obesogenic environment was associated with hypertrophic remodeling of the mesenteric resistance vasculature in juvenile mice offspring. This was manifested as an increased media to lumen ratio of the arteries and an augmented cross sectional area of the vascular wall. Although we observed no significant changes in passive luminal diameter in association with the obesogenic environment, overnutrition in mice has been previously associated with outward hypertrophic remodeling in db/db mice [29]. In the latter study, outward remodeling was deemed to have occurred as a consequence of persistent increased flow-induced vasodilation, which is NO dependent. Therefore, it is plausible that the increased vascular oxidative stress we found in the arteries of ART mice prevented the development of outward remodeling associated with the WD in our current study. The observation that endothelium-independent vasodilatory responses to SNP were not affected by WD or ART suggests that both the functional and structural changes we found in mesenteric resistance arteries are likely associated to vascular endothelial dysfunction.

The literature consistently indicates that, early in life, males are more susceptible than females to develop endothelial dysfunction and hypertension [30]. Accordingly we found that male mice had increased vasoconstrictor responses to phenylephrine and reduced vasodilator responses to acetylcholine compared to females. These results are consistent with our previous report in which we showed that these same male mice had greater mean arterial pressures than females at seven weeks of age [18].

Developmental programming of diseases such as hypertension and diabetes has been associated with oxidative stress. Our current finding that ART reduced the vasodilatory capacity of resistance arteries from mice in the WD group suggests that an interaction exists between an obesogenic environment and the use of ART to induce endothelial dysfunction. Our previous finding that the use of ART is associated with an increased level of ROS in the wall of resistance vessels further suggests that the mechanism responsible for this interaction may very well be oxidative stress. At this time there is no clear evidence on which mechanism(s) may be responsible for increasing ROS in the resistance vessels of ART mice and why only ART mice in the WD group showed functional endothelial dysfunction. We previously showed that the expression of the NADPH oxidase, NOX2, in the resistance arteries of these mice is not affected by ART or diet [18]. Our current results indicating that there is a marginal reduction of SOD1 in ART mice suggest that ART may cause a reduction in vascular antioxidant capacity. Whether this reduction in SOD1 expression is sufficient to functionally increase vascular oxidation remains to be determined.

As we observed that the use of ART increased vascular ROS, and as obesity and diabetes are commonly accompanied by oxidative stress [31], we probed the plasma of mice for a number of biomarkers commonly associated with glucose metabolism, diabetes or diabetes development. Not surprisingly, the obesogenic environment caused by feeding dams and offspring a WD resulted in increased plasma concentrations of GIP, GLP-1, insulin, leptin, and resistin. GIP and GLP-1 are incretins, gastrointestinal peptide hormones that increase insulin secretion in response to meal intake. Consistent with our findings, previous reports have indicated that both GIP and GLP-1 levels are increased in response to high-fat obesogenic diets [32], [33]. An overwhelming body of literature also associates obesogenic WDs with hyperinsulinemia, hyperleptinemia, and insulin resistance [31], [34], [35]. As leptin is an anorexic cytokine produced by adipose tissue, it is considered that the hyperleptinemia observed in obese subjects is associated with reduced leptin sensitivity [36], [37]. Contrary to our expectations, no significant interactions between diet and ART were observed to affect these biomarkers.

Reports on the levels of circulating resistin found in individuals with obesity and T2DM is controversial [38], but it is clear that resistin is associated with reduced insulin sensitivity [39], [40]. Consequently, our finding that resistin levels were increased in WD fed mice is consistent with our observation that these mice were also hyperinsulinemic. We also found a significant diet x ART interaction indicating that the increased levels of resistin found in the WD fed mice were greater in animals obtained by ART than in those obtained via natural conception, gestation, and birth. It remains to be determined whether the association between ART and an obesogenic environment had any mechanistic relation with the increased levels of oxidative stress we found in the resistance vessels of ART mice.

Significant diet x sex interactions were observed for leptin and PAI-1. Leptin was significantly greater in males vs. female only in mice fed a WD. As mentioned above WD was associated with greater leptin levels in both sexes. In comparison, PAI-1 levels were significantly greater in males vs. females only in mice fed CD. Consumption of a WD increased PAI-1 serum concentrations only in females. Male offspring have been shown to be more severely programmed for obesity and cardiovascular disease than females when exposed to a maternal obesogenic environment [41], [42]. Our observation that leptin levels increased more in males than in females when fed a WD is in accord with those reports. As for the differential effect of a WD on PAI-1 levels in male and females, our results suggest that a WD increased PAI-1 concentrations only in females. Previously, PAI-1 concentrations have been found to be correlated with body mass index, which is an indirect measure of adiposity in humans [43], [44]. We did not measure adiposity in our current study, but we have previously reported that both male and female mice fed the WD were significantly heavier than those fed the CD [18]. Whether this increase in PAI-1 concentration in female mice fed a WD translates into a more prothrombotic phenotype remains to be determined. Additional studies are also needed to determine if the trend for an ART x diet interaction (P = 0.06) we observed for PAI-1 affects thrombosis. That trend suggests that the use of ART may increase PAI-1 concentrations only in mice exposed to an obesogenic environment.

Our observation that plasma ghrelin was increased in mice fed a WD is contradictory to previous results indicating diet-induced obesity in mice is associated with low plasma levels of ghrelin [45], [46], but consistent with the enlarged body weight of these mice. Increased serum levels of ghrelin are also contrary to the increased levels of insulin, leptin and resistin we found, as previous reports indicate ghrelin reduces insulin secretion and is negatively correlated with leptin and resistin [47], [48], [49], [50]. However, it is important to consider that the assay we used in this study measured total ghrelin and not only the active acylated form of this gastric peptide hormone [51]. Additional experiments will be needed to investigate the level of the acylated form of ghrelin, as well as the other measured biomarkers in fasted animals and at different times during feed intake and the development of obesity.

An important limitation of our study resides in the fact that our experimental design was not developed to interrogate the differential effects of exposure to an obesogenic environment during fetal development vs. exposure after birth. This study was designed to investigate the interaction between the use of ART and an obesogenic environment as it occurs in obese women using ART. An additional limitation of our study is that our experimental design does not allow for identification of the independent contributions of each of the components of our ART protocol in mice to specific effects attributed to ART on the measurements obtained in the offspring [52]. We have used the term ART in this study in reference to all three procedures combined in our protocol namely, superovulation, embryo culture and embryo transfer. Future experiments will be developed to determine the specific effects of each of these procedures on the offspring. Our major conclusion is that ART, as defined in this study, interacts with the presence of an obesogenic environment to reduce the capacity of mesenteric resistance arteries to vasodilate in response to acetylcholine stimulation and to become remodeled. Further studies will be required to determine whether these interactions will influence the development of cardiovascular disease later in life.

## Supporting Information

Figure S1 **Effect of diet and ART on the passive properties of resistance arteries.**
(PDF)

Figure S2 **Effect of diet and ART on serum PAI-1 concentrations.**
(PDF)

Figure S3 **Effect of ART on arterial SOD-1 expression.**
(PDF)

Table S1 **Offspring distribution per surrogate dam.**
(PDF)

Methods S1 **Extended version of the statistical analysis.**
(PDF)
