# Supplementary material for: Effects of the Use of Assisted Reproductive Technologies and an Obesogenic Environment on Resistance Artery Function and Diabetes Biomarkers in Mice Offspring
Source: PLoS One. 2014 Nov 11;9(11):e112651. doi: 10.1371/journal.pone.0112651 (PMC4227714; doi:10.1371/journal.pone.0112651)
Supplement: Figure S1 — Effect of diet and ART on the passive properties of resistance arteries. (PDF) [file pone.0112651.s001.pdf]

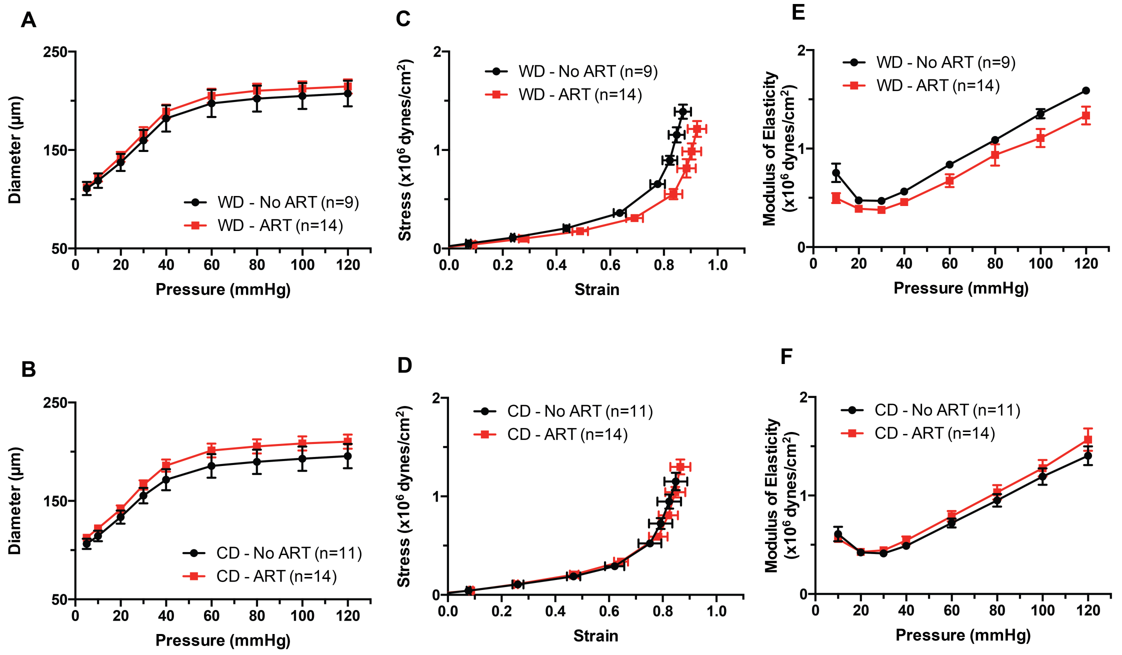

Figure S1. Effect of diet and ART on the passive diameter and elastic properties of mesenteric resistance arteries from juvenile mice. (A,B) Maximal passive diameters under calcium-free conditions at different intravascular pressures in isolated resistance arteries. (C,D) Strain-stress relationships of isolated mesenteric resistance arteries under passive conditions. (E,F) Moduli of elasticity of isolated mesenteric resistance vessels at different intravascular pressures under passive conditions. Data are means ± SEM.
