# Supplementary material for: Effects of the Use of Assisted Reproductive Technologies and an Obesogenic Environment on Resistance Artery Function and Diabetes Biomarkers in Mice Offspring
Source: PLoS One. 2014 Nov 11;9(11):e112651. doi: 10.1371/journal.pone.0112651 (PMC4227714; doi:10.1371/journal.pone.0112651)
Supplement: Figure S2 — Effect of diet and ART on serum PAI-1 concentrations. (PDF) [file pone.0112651.s002.pdf]

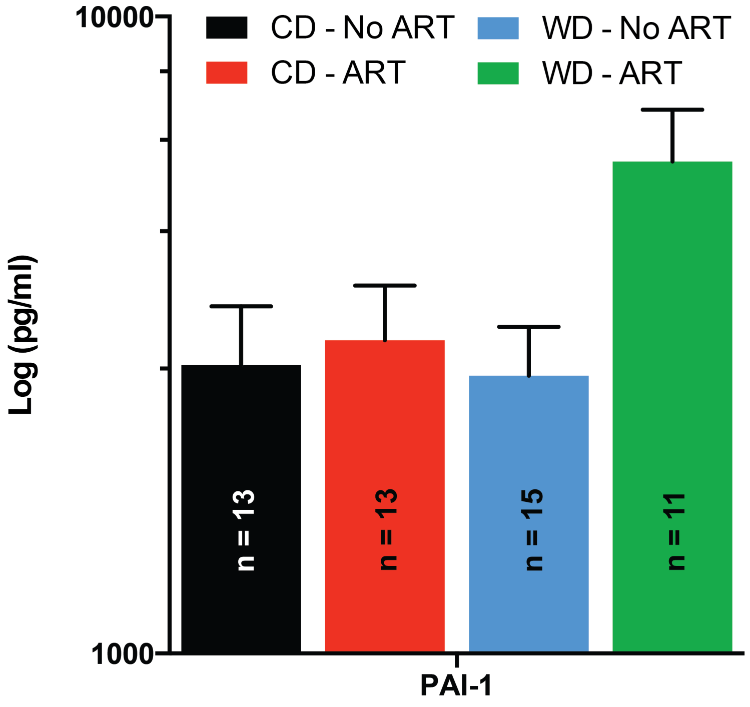

Figure S2. Effect of diet and ART on serum PAI-1 concentrations in juvenile mice. Serum PAI-1 concentrations were numerically greater (P=0.06) in WD-ART than in in WD-No ART mice. Data are means ± SEM.
