# Supplementary material for: Effects of the Use of Assisted Reproductive Technologies and an Obesogenic Environment on Resistance Artery Function and Diabetes Biomarkers in Mice Offspring
Source: PLoS One. 2014 Nov 11;9(11):e112651. doi: 10.1371/journal.pone.0112651 (PMC4227714; doi:10.1371/journal.pone.0112651)
Supplement: Figure S3 — Effect of ART on arterial SOD-1 expression. (PDF) [file pone.0112651.s003.pdf]

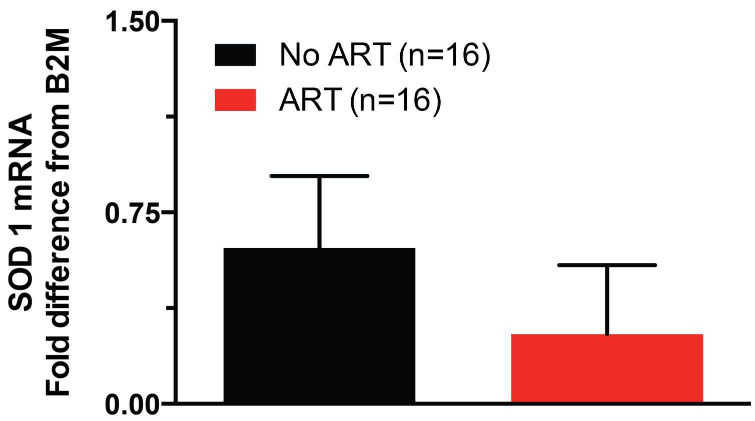

Figure S3. Effect of ART on expression of SOD-1 in juvenile mice. Quantitative RT-PCR assessment of expression of the SOD-1 gene in mice blood vessels. Gene expression was normalized to the expression of B2m (DCT) and then averaged within the respective groups and expressed as fold difference. Data are means ± SEM. qRT-PCR procedures were as described in Schenewerk et al., and TaqMan probes for SOD1 and B2m were purchased from Applied Biosystems (Assay ID- Mm01344233_g1 and Mm00430072_m1, respectively).
