# Supplementary material for: Effects of the Use of Assisted Reproductive Technologies and an Obesogenic Environment on Resistance Artery Function and Diabetes Biomarkers in Mice Offspring
Source: PLoS One. 2014 Nov 11;9(11):e112651. doi: 10.1371/journal.pone.0112651 (PMC4227714; doi:10.1371/journal.pone.0112651)
Supplement: Table S1 — Offspring distribution per surrogate dam. (PDF) [file pone.0112651.s004.pdf]

Table S1: Offspring Distribution Per Dam

Vascular experiments

	No ART CD				No ART WD				ART CD				ART WD			
	Dam	Male offspring	Dam	Female offspring	Dam	Male offspring	Dam	Female offspring	Dam	Male offspring	Dam	Female offspring	Dam	Male offspring	Dam	Female offspring
	D1	2	D1	1	D1	1	D1	2	D1	3	D1	1	D1	4	D1	2
	D2	1	D2	1	D2	2	D2	1	D2	1	D2	3	D2	4	D2	3
	D3	1	D3	2	D3	1	D3	1	D3	1	D3	2	D3	1		
	D4	2	D4	2	D4	2	D4	2	D4	2	D4	1				
									D5	1						
	Dams	Offspring	Dams	Offspring	Dams	Offspring	Dams	Offspring	Dams	Offspring	Dams	Offspring	Dams	Offspring	Dams	Offspring
**Total**	**4**	**6**	**4**	**6**	**4**	**6**	**4**	**6**	**5**	**8**	**4**	**7**	**3**	**9**	**2**	**5**

Bio-Plex experiments

	No ART CD				No ART WD				ART CD				ART WD			
	Dam	Male offspring	Dam	Female offspring	Dam	Male offspring	Dam	Female offspring	Dam	Male offspring	Dam	Female offspring	Dam	Male offspring	Dam	Female offspring
	D1	2	D1	1	D1	2	D1	2	D1	1	D1	1	D1	4	D1	2
	D2	1	D2	1	D2	2	D2	1	D2	1	D2	3	D2	3	D2	3
	D3	1	D3	2	D3	2	D3	1	D3	1	D3	3	D3	1		
	D4	1	D4	1	D4	2	D4	1	D4	4	D4	1				
	D5	2	D5	1			D5	2	D5	1						
			D6	2												
	Dams	Offspring	Dams	Offspring	Dams	Offspring	Dams	Offspring	Dams	Offspring	Dams	Offspring	Dams	Offspring	Dams	Offspring
**Total**	**5**	**7**	**6**	**8**	**4**	**8**	**5**	**7**	**5**	**8**	**4**	**8**	**3**	**8**	**2**	**5**

This Table describes the distribution of offspring obtained from different surrogate dams and how they were included in experiments related to vascular structure and function, and those related to the measurement of diabetes biomarkes using the Bio-Plex system.
