# Supplementary material for: Effects of the Use of Assisted Reproductive Technologies and an Obesogenic Environment on Resistance Artery Function and Diabetes Biomarkers in Mice Offspring
Source: PLoS One. 2014 Nov 11;9(11):e112651. doi: 10.1371/journal.pone.0112651 (PMC4227714; doi:10.1371/journal.pone.0112651)
Supplement: Methods S1 — Extended version of the statistical analysis. (PDF) [file pone.0112651.s005.pdf]

Statistical Analysis

Data Analysis for Artery Diameter Change with the Application of Vasoactive Agents

Before model fitting, samples that did not constrict at least 20% relative to the maximal passive diameter with the application of high K^+ were removed from analysis. For each of the three applications of vasoactive agonists (phenylephrine, acetylcholine, and SNP), samples that did not constrict at least 10% relative to the maximal diameter with the initial application of phenylephrine were removed from the analysis of that vasoactive agent.

The offspring arterial diameters were modeled individually to determine which factors were significant when testing the elasticity of the sample with each of the vasoactive agents: phenylephrine, acetylcholine, and SNP. The diameters were normalized to represent a percent relative to their maximal diameter and initial constriction to account for the large variability amongst the arterial diameters. These percentages were regressed to the ART effect (pregnancy through ART or No ART), diet effect (WD or CD), sex effect (female or male), and concentration of the vasoactive agents (over time) as well as interaction effects between each two, three, and four factors. Mothers were included as random effects in the model to account for the correlation between offspring of the same litter. Offspring were included as random effects to account for the correlation between two arteries extracted from the same offspring. Repeated measurements of one same artery of an offspring were taken at several time points for each vasoactive agent. Thus, to account for the correlation among the repeated measurements, a heterogeneous compound symmetry correlation structure was used to model the correlation between these time points. This correlation structure was chosen for modeling the repeated measurements based on its AIC and BIC statistics as well as its reasonable biological interpretation. Each model was constructed using backwards elimination by statistical significance. The main effects and interaction effects were selected by controlling the statistical significance level at 0.10. The studentized residual plots for each of the models suggest that the normality assumption is met in all cases. The Kenward-Roger adjustment to denominator degrees of freedom was used when fitting all three models in this study because of the

unbalanced design, random effects, repeated measures, and moderate sample size. After model selection, the Tukey-Kramer HSD method was used to adjust multiple tests in comparing different levels of treatment combinations.

Data Analysis for Plasma Measurements of Diabetes Associated Biomarkers

A model was fit to each of the eight biomarkers based on ART effect (pregnancy through ART or No ART), diet effect (WD or CD), sex effect (female or male). Again, mothers were included as random effects to account for correlation of offspring from the same mother. We also examined interactions of ART, diet effect, and sex effect of the offspring. The main effects and interaction effects were selected by controlling the significance level at 0.10. The Kenward-Roger adjustment to denominator degrees of freedom was used when fitting each protein model. Influence measure diagnostics were calculated for each observation, and highly influential mouse replicates were removed from the analysis based on their DFFITS and Cook's Distance values. Transformations were applied to maintain normality and constant variance model assumptions when necessary. Tukey-Karmer HSD model was applied to adjust multiple tests in pair wise comparisons.
